# Supplementary material for: Identification of the WRKY Gene Family and Characterization of Stress-Responsive Genes in Taraxacum kok-saghyz Rodin
Source: Int J Mol Sci. 2022 Sep 7;23(18):10270. doi: 10.3390/ijms231810270 (PMC9499643; doi:10.3390/ijms231810270)
Supplement: Supplementary file 1 [file ijms-23-10270-s001.zip › Table S1 TKS WRKY genes and legends.pdf]

**Table S1** TKS WRKY genes and legends.

| Protein name | Accession number | Group | pI(isoelectric point) | Molecular weight | Amino acid | Aliphatic index | Instability index | GRAVY | Subcellular localization |
|---|---|---|---|---|---|---|---|---|---|
| *TkWRKY1* | GWHPAAAA001298 | II-e | 6.09 | 55473.21 | 502 | 65.08 | 54.52 | -0.62 | Mitochondrion |
| *TkWRKY2* | GWHPAAAA001365 | I | 9.5 | 39669.16 | 355 | 69.38 | 41.28 | -0.734 | Nucleus |
| *TkWRKY3* | GWHPAAAA001899 | II-c | 6.26 | 31221.02 | 280 | 59.25 | 52.62 | -0.641 | Nucleus |
| *TkWRKY4* | GWHPAAAA002041 | II-c | 5.4 | 40024.98 | 363 | 53.17 | 63.47 | -0.934 | Nucleus |
| *TkWRKY5* | GWHPAAAA003686 | II-b | 6.99 | 53916.69 | 495 | 64.26 | 47.28 | -0.708 | Nucleus |
| *TkWRKY6* | GWHPAAAA004870 | II-e | 9.78 | 29421.19 | 274 | 62.3 | 53.64 | -0.501 | Nucleus |
| *TkWRKY7* | GWHPAAAA005167 | II-d | 9.68 | 38246.18 | 343 | 69.39 | 47.4 | -0.799 | Nucleus |
| *TkWRKY8* | GWHPAAAA005780 | II-e | 10.07 | 7743.15 | 67 | 58.21 | 49.58 | -0.918 | Nucleus |
| *TkWRKY9* | GWHPAAAA008417 | II-e | 9.39 | 14609.24 | 128 | 38.05 | 63.2 | -1.207 | Nucleus |
| *TkWRKY10* | GWHPAAAA008486 | III | 5.58 | 37007.24 | 325 | 62.65 | 63.62 | -0.794 | Nucleus |
| *TkWRKY11* | GWHPAAAA008502 | I | 6.51 | 34672.21 | 311 | 72.67 | 52.77 | -0.622 | Nucleus |
| *TkWRKY12* | GWHPAAAA008804 | II-b | 5.97 | 56293.68 | 515 | 70.12 | 39.47 | -0.597 | Nucleus |
| *TkWRKY13* | GWHPAAAA008866 | I | 9.13 | 25773.12 | 224 | 66.38 | 53.55 | -0.823 | Nucleus |
| *TkWRKY14* | GWHPAAAA008929 | II-b | 8.71 | 48320.03 | 437 | 61.62 | 57.08 | -0.765 | Nucleus |
| *TkWRKY15* | GWHPAAAA009771 | III | 6.04 | 34194.88 | 304 | 67.63 | 53.71 | -0.647 | Nucleus |
| *TkWRKY16* | GWHPAAAA010357 | II-c | 9.77 | 8944.06 | 75 | 40.13 | 37.54 | -1.361 | Nucleus |
| *TkWRKY17* | GWHPAAAA010789 | II-b | 7.73 | 57932.3 | 532 | 67.71 | 43.52 | -0.636 | Nucleus |
| *TkWRKY18* | GWHPAAAA011100 | I | 6.24 | 54327.16 | 491 | 44.89 | 50.92 | -0.979 | Nucleus |
| *TkWRKY19* | GWHPAAAA012630 | II-b | 6.8 | 60183.92 | 552 | 62.45 | 62.24 | -0.687 | Nucleus |
| *TkWRKY20* | GWHPAAAA012956 | II-c | 5.39 | 41648.12 | 362 | 78.65 | 50.56 | -0.52 | Nucleus |
| *TkWRKY21* | GWHPAAAA013629 | II-b | 7.6 | 60587.07 | 556 | 55.63 | 48.55 | -0.784 | Nucleus |
| *TkWRKY22* | GWHPAAAA014488 | II-d | 9.69 | 37328.3 | 335 | 68.69 | 53.23 | -0.749 | Nucleus |
| *TkWRKY23* | GWHPAAAA014696 | II-c | 5.12 | 33974.43 | 305 | 54 | 61.36 | -1.032 | Nucleus |
| *TkWRKY24* | GWHPAAAA015131 | II-c | 6.58 | 24626.42 | 213 | 49.86 | 38.32 | -0.881 | Nucleus |
| *TkWRKY25* | GWHPAAAA016783 | I | 6.96 | 53981.32 | 481 | 52.66 | 58.18 | -0.965 | Nucleus |
| *TkWRKY26* | GWHPAAAA017465 | II-b | 5.94 | 57060.31 | 526 | 66.12 | 47.06 | -0.635 | Nucleus |
| *TkWRKY27* | GWHPAAAA018006 | III | 8.81 | 31061.44 | 281 | 73.17 | 63.92 | -0.449 | Nucleus |
| *TkWRKY28* | GWHPAAAA018231 | II-c | 9.21 | 21926.23 | 193 | 53.47 | 41.05 | -1.045 | Nucleus |
| *TkWRKY29* | GWHPAAAA018450 | I | 7.22 | 53191.52 | 475 | 52.51 | 52.67 | -0.98 | Nucleus |
| *TkWRKY30* | GWHPAAAA018833 | II-c | 9.32 | 20486.79 | 181 | 58.23 | 40.98 | -0.887 | Nucleus |
| *TkWRKY31* | GWHPAAAA018955 | I | 7.38 | 54953.3 | 504 | 50.67 | 55.94 | -0.918 | Nucleus |
| *TkWRKY32* | GWHPAAAA019110 | III | 6.07 | 30136.95 | 264 | 69.39 | 53.89 | -0.716 | Nucleus |
| *TkWRKY33* | GWHPAAAA019111 | III | 9.6 | 42014.17 | 371 | 64.72 | 54.28 | -0.772 | Nucleus |
| *TkWRKY34* | GWHPAAAA019406 | II-b | 8.19 | 48299.75 | 436 | 56.38 | 48.25 | -0.746 | Nucleus |
| *TkWRKY35* | GWHPAAAA020203 | I | 7.37 | 55103.44 | 496 | 53.23 | 43.82 | -0.996 | Nucleus |
| *TkWRKY36* | GWHPAAAA022971 | I | 9.23 | 39159.9 | 352 | 58.95 | 52.84 | -0.924 | Nucleus |
| *TkWRKY37* | GWHPAAAA024558 | II-e | 9.94 | 12003.34 | 106 | 40.38 | 62.27 | -1.354 | Nucleus |
| *TkWRKY38* | GWHPAAAA024882 | I | 6.95 | 58043.72 | 520 | 54.19 | 62.87 | -0.915 | Nucleus |
| *TkWRKY39* | GWHPAAAA024953 | III | 6.05 | 37454.9 | 337 | 62.52 | 57.98 | -0.747 | Nucleus |
| *TkWRKY40* | GWHPAAAA025085 | II-b | 6.12 | 62383.85 | 576 | 60.87 | 54.99 | -0.694 | Nucleus |
| *TkWRKY41* | GWHPAAAA025108 | II-c | 6.08 | 20876.11 | 184 | 51.9 | 42.14 | -0.872 | Nucleus |

| | | | | | | | | | |
|---|---|---|---|---|---|---|---|---|---|
| *TkWRKY42* | GWHPAAAA026343 | I | 5.81 | 75095.86 | 676 | 56.21 | 48.73 | -0.897 | Nucleus |
| *TkWRKY43* | GWHPAAAA026852 | III | 5.71 | 36819.05 | 324 | 62.84 | 62.84 | -0.8 | Nucleus |
| *TkWRKY44* | GWHPAAAA027845 | I | 9.51 | 39709.23 | 355 | 70.48 | 43.24 | -0.721 | Nucleus |
| *TkWRKY45* | GWHPAAAA028118 | III | 9.01 | 30354.31 | 265 | 74.6 | 49.89 | -0.65 | Nucleus |
| *TkWRKY46* | GWHPAAAA028318 | II-c | 8.7 | 30508.12 | 276 | 52.97 | 68.4 | -0.6 | Nucleus |
| *TkWRKY47* | GWHPAAAA028692 | I | 5.78 | 28812.42 | 261 | 69.08 | 49.38 | -0.644 | Nucleus |
| *TkWRKY48* | GWHPAAAA028846 | I | 6.18 | 71785.26 | 655 | 45.25 | 56.08 | -1.005 | Nucleus |
| *TkWRKY49* | GWHPAAAA029779 | II-c | 6.39 | 32822.05 | 292 | 59.49 | 52.81 | -0.632 | Nucleus |
| *TkWRKY50* | GWHPAAAA029797 | II-e | 5.36 | 24552.93 | 221 | 50.68 | 47.2 | -0.784 | Nucleus |
| *TkWRKY51* | GWHPAAAA030787 | II-d | 9.73 | 37286.05 | 345 | 65.07 | 46.33 | -0.507 | Nucleus |
| *TkWRKY52* | GWHPAAAA031944 | II-a | 6.1 | 29439.1 | 259 | 71.47 | 48.54 | -0.782 | Nucleus |
| *TkWRKY53* | GWHPAAAA032696 | II-b | 5.73 | 55035.11 | 494 | 63.18 | 57.35 | -0.843 | Nucleus |
| *TkWRKY54* | GWHPAAAA033671 | none | 4.88 | 17926.68 | 162 | 49.32 | 46.63 | -0.89 | Nucleus |
| *TkWRKY55* | GWHPAAAA034498 | II-c | 7.86 | 20010.4 | 179 | 71.28 | 45.56 | -0.703 | Nucleus |
| *TkWRKY56* | GWHPAAAA034566 | II-d | 9.56 | 34565.25 | 318 | 66.86 | 52 | -0.574 | Nucleus |
| *TkWRKY57* | GWHPAAAA034574 | II-c | 9.44 | 23142.24 | 199 | 58.29 | 47.48 | -0.879 | Nucleus |
| *TkWRKY58* | GWHPAAAA035988 | II-c | 6.3 | 20402.44 | 179 | 52.79 | 50.41 | -0.991 | Nucleus |
| *TkWRKY59* | GWHPAAAA036147 | III | 5.31 | 25236.83 | 224 | 70.04 | 41.96 | -0.703 | Nucleus |
| *TkWRKY60* | GWHPAAAA036334 | III | 5.27 | 16835.05 | 150 | 77.27 | 34.28 | -0.546 | Nucleus |
| *TkWRKY61* | GWHPAAAA036496 | II-c | 9.08 | 23805.67 | 211 | 66.45 | 44.62 | -0.84 | Nucleus |
| *TkWRKY62* | GWHPAAAA036499 | II-e | 5.68 | 37602.5 | 331 | 59.58 | 54.83 | -0.86 | Nucleus |
| *TkWRKY63* | GWHPAAAA036778 | II-d | 9.69 | 37012.48 | 340 | 60.26 | 51.25 | -0.634 | Nucleus |
| *TkWRKY64* | GWHPAAAA037940 | I | 8.51 | 53630.8 | 487 | 58.42 | 65.08 | -0.803 | Nucleus |
| *TkWRKY65* | GWHPAAAA039332 | II-c | 6.66 | 33595.31 | 301 | 51.43 | 59.55 | -0.81 | Nucleus |
| *TkWRKY66* | GWHPAAAA040169 | II-c | 8.28 | 33211.78 | 303 | 56.86 | 47.11 | -0.751 | Nucleus |
| *TkWRKY67* | GWHPAAAA040219 | I | 8.26 | 42201.99 | 379 | 63.27 | 51.26 | -0.921 | Nucleus |
| *TkWRKY68* | GWHPAAAA041256 | II-e | 6.45 | 35636.43 | 320 | 47.88 | 51.43 | -0.962 | Nucleus |
| *TkWRKY69* | GWHPAAAA041614 | II-b | 8.78 | 43809.39 | 394 | 67.13 | 47.5 | -0.635 | Nucleus |
| *TkWRKY70* | GWHPAAAA042782 | III | 7.82 | 16604.73 | 150 | 85.2 | 46.37 | -0.526 | Nucleus |
| *TkWRKY71* | GWHPAAAA043806 | II-a | 8.37 | 36300.67 | 329 | 66.35 | 41.22 | -0.728 | Nucleus |
| *TkWRKY72* | GWHPAAAA046275 | III | 5.1 | 33931.8 | 301 | 66.74 | 66.2 | -0.619 | Nucleus |

**Table S2** The 20 motifs in *Taraxacum kok-saghyz* Rodin WRKY proteins.

| Motif number | Motif Sequence | Motif Logo | Width |
|---|---|---|---|
| Motif 1 | DGYRWRKYGQKVVKG | 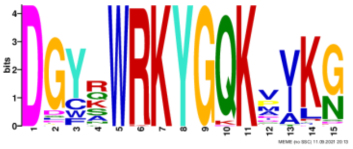 | 15 |
| Motif 2 | SPHPRSYYRCTSAGC | 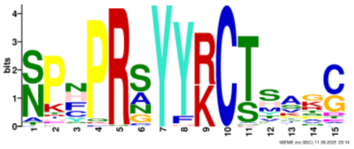 | 15 |

| | | | |
|---|---|---|---|
| Motif 3 | DPSIVITTYEGKHNH | 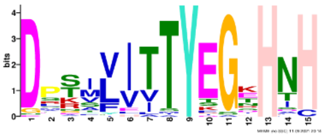 | 15 |
| Motif 4 | DDGYNWRKYGQKQVKGS EYPRSYYKCTHPNCPVKK | 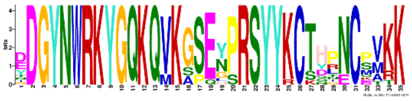 | 35 |
| Motif 5 | KKIREPRVAVQTRSEVDILD | 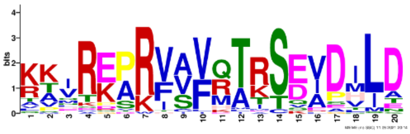 | 20 |
| Motif 6 | PVRKQVZRASD | 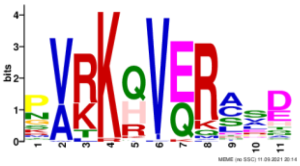 | 11 |
| Motif 7 | GQITEIVYKGNHNHPKPQPTK | 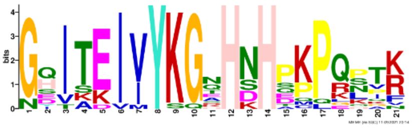 | 21 |
| Motif 8 | PLPPAATAMASTTSAAASMLL | 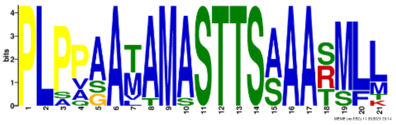 | 21 |
| Motif 9 | VQAELERMNEENQRLREMLDQV MKBYNALQMHLKTVMQQKQQEE | 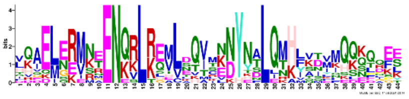 | 44 |
| Motif 10 | ALAETTTAJTADPNFTAALAAAISS IIGG | 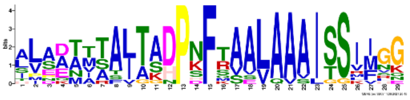 | 29 |
| Motif 11 | SSGRCHCSKKRKHRMKR VVRVPAISMKLADIPP | 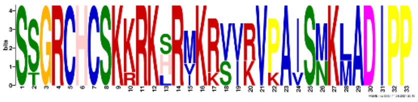 | 33 |
| Motif 12 | GJSPSNFLESPFLLTNSSIAPSPTSGS FP | 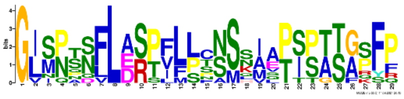 | 29 |
| Motif 13 | GFGTGISPGPMTLVSNFFSDHYPD ADLRSFSQLLAGVIPSPAAEYPPPH C | 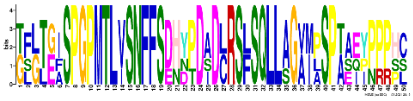 | 50 |
| Motif 14 | KKKLIEELTQGQELAKKLKRLLRR PQSSE | 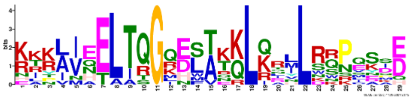 | 29 |

| | | | |
|---|---|---|---|
| Motif 15 | GGGGGGGFFDTPQGYLDMLAFQDYGGASLFDLLQQPTPPNLVVEQLQIH | 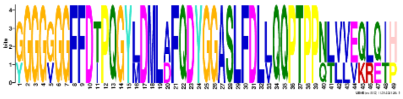 | 49 |
| Motif 16 | LVDEILGSFKKALSLLNST | 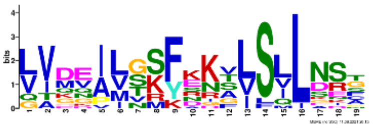 | 19 |
| Motif 17 | VTPVKERRGCYKRRKTSDSRVKIAPTIE | 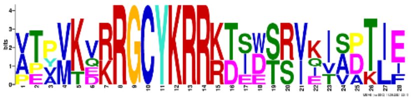 | 28 |
| Motif 18 | PFMDHGLAPPSLVEDEEIPTSSADGHDEE | 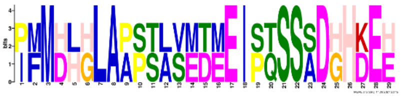 | 29 |
| Motif 19 | PTTIQFPLNLNCNHDHQEKQINEMDFFSDKKHDEDRSRLPV | 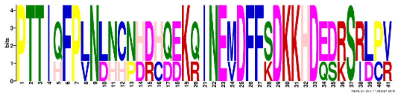 | 41 |
| Motif 20 | PTRIPYFNYSLNPTTYAPAMDHGGLLQDVLAYHRQGTPADH | 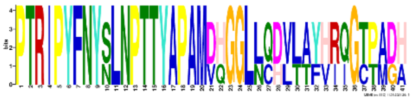 | 41 |

Note: In Motif Logo, the larger the letter, the more conserved the amino acid.

**Table S3** The 20 motifs in nine species (rice, maize, *Brachypodium distachyon*, pineapple, *Arabidopsis*, grapes, peaches, poplars and *Taraxacum kok-saghyz* Rodin) WRKY proteins.

| Motif number | Motif Sequence | Motif Logo | Width |
|---|---|---|---|
| Motif 1 | PIDDGYRWRKYGQKHILGAKHPRSYYRC | 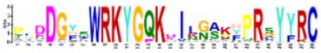 | 28 |
| Motif 2 | DPHLFLYTYRGLHTC | 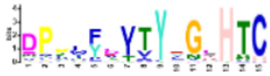 | 15 |
| Motif 3 | DQGCTATKQVQRSDD | 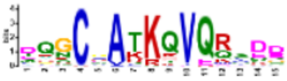 | 15 |
| Motif 4 | LYLELYRGKELAKQLETLLRP | 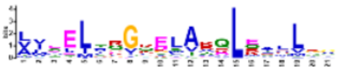 | 21 |
| Motif 5 | ERVELLVQKILSSFERALSILN | 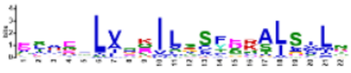 | 22 |
| Motif 6 | KKRKTLPRWTEQVRV | 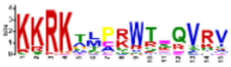 | 15 |

| | | | |
|---|---|---|---|
| Motif 7 | VKDRRGCYKRRKTSQSWTTV | 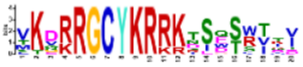 | 20 |
| Motif 8 | ESDLTEIISAATSVTNSPIVD | 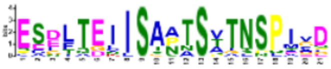 | 21 |
| Motif 9 | PSFISPATSESNYFSVSPCQM | 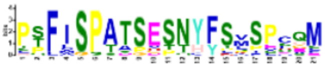 | 21 |
| Motif 10 | AQEHNPQAQGLLQSLQAGLTVKTEGLD | 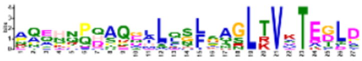 | 27 |
| Motif 11 | LDDGYRWRKYGQKVVKGNPRSYYKCT | 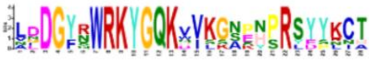 | 28 |
| Motif 12 | YVDLADAMFNSGSSSTN | 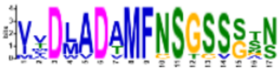 | 17 |
| Motif 13 | VEFDPNFPFDNPGFF | 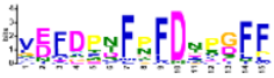 | 15 |
| Motif 14 | SSSQRPRRRKDEGERRTYRVPAQRTGNTEI | 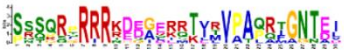 | 30 |
| Motif 15 | KAFAVHDDTYGSEMIKFDQVVSESVMPQLATI DEQAITMED | 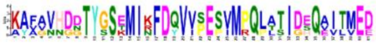 | 41 |
| Motif 16 | HIMNQECDINDYLVDDDPFWASQFPPF | 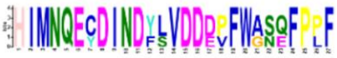 | 27 |
| Motif 17 | SPNGSLSTHVASYPVFEYAEWRGDADLQEVVS | 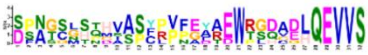 | 32 |
| Motif 18 | LPSSSIIIPFFSTPCTSNSNQPQKNNYFTESVISNL VMTGF | 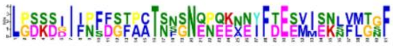 | 41 |
| Motif 19 | SPSSGNGSPRSEDSD | 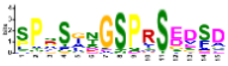 | 15 |
| Motif 20 | SGLDHGDVISSVNYSSTTSTHSYDIDNM | 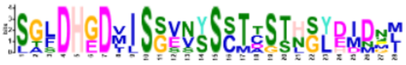 | 28 |

Note: In Motif Logo, the larger the letter, the more conserved the amino acid.

**Table S4** Statistics of *cis*-acting elements of TkWRKY genes.

| Gene name | WUN-motif | MBS | LTR | MBSI | W-box | TC-rich repeats | CGTCA-motif | ABRE | TCA-element | TGA-element | GARE-motif | circadian |
|---|---|---|---|---|---|---|---|---|---|---|---|---|

| | | | | | | | | | | | | |
|---|---|---|---|---|---|---|---|---|---|---|---|---|
| *TkWRKY1* | 1 | 1 | | | 1 | 1 | 3 | 1 | | | | |
| *TkWRKY2* | 1 | 1 | 1 | | 1 | | 2 | 1 | | 1 | | |
| *TkWRKY3* | | 1 | 2 | | 1 | | 2 | 1 | | 2 | 1 | |
| *TkWRKY4* | | | 1 | | 2 | 1 | 3 | 2 | | | | 1 |
| *TkWRKY5* | 1 | 1 | | | 2 | 1 | 1 | 2 | 3 | | | |
| *TkWRKY6* | | | 5 | | | 1 | 3 | 1 | | | | 1 |
| *TkWRKY7* | 1 | | | | 2 | 1 | 3 | | | 1 | | |
| *TkWRKY8* | | 1 | | | 1 | | 4 | 1 | | 2 | | |
| *TkWRKY9* | | | 1 | | 1 | | 1 | 1 | | 2 | | 1 |
| *TkWRKY10* | | 1 | 1 | | 3 | | 1 | 5 | | | | |
| *TkWRKY11* | 1 | 1 | 2 | | | | | 1 | 2 | 2 | | |
| *TkWRKY12* | 1 | | | | 1 | | 5 | 5 | 2 | | | |
| *TkWRKY13* | 1 | 1 | 1 | | 1 | | 2 | 1 | 1 | 2 | | |
| *TkWRKY14* | | 1 | 3 | | 1 | 1 | 3 | 3 | | 1 | | |
| *TkWRKY15* | 1 | 1 | 2 | | | | 4 | 3 | 2 | 1 | | |
| *TkWRKY16* | 1 | 1 | | | | | 1 | 3 | 1 | | | |
| *TkWRKY17* | 1 | 1 | 1 | | 1 | 1 | 2 | 2 | 1 | | | |
| *TkWRKY18* | | 2 | 2 | | 2 | | 1 | | 1 | 1 | 1 | 1 |
| *TkWRKY19* | | 2 | 1 | | 2 | | 3 | 2 | | | | |
| *TkWRKY20* | | | | | 2 | 1 | | 1 | | | | |
| *TkWRKY21* | 4 | 1 | 1 | | | | | | 1 | | 1 | |
| *TkWRKY22* | 1 | 1 | 1 | | | | 6 | 2 | | 1 | | 1 |
| *TkWRKY23* | | | | | 2 | 2 | 3 | 2 | | | | |
| *TkWRKY24* | 4 | | | | 3 | | 3 | 1 | 2 | | | |
| *TkWRKY25* | | | | | | | 5 | 2 | | | | |
| *TkWRKY26* | 2 | | | | 1 | 1 | 1 | 7 | | | | |
| *TkWRKY27* | 1 | 4 | 1 | | | | 3 | 1 | | | | |
| *TkWRKY28* | 1 | | | | 5 | | | 2 | 1 | | | |
| *TkWRKY29* | | | 1 | 1 | | 1 | 2 | 4 | | | | |
| *TkWRKY30* | | 1 | | | 2 | | 3 | 3 | | | | 1 |
| *TkWRKY31* | 1 | | | | 1 | | | 4 | | | | |
| *TkWRKY32* | 1 | | 1 | | 1 | 2 | 3 | 1 | | | | |
| *TkWRKY33* | | 1 | 2 | | | | 2 | 1 | | 1 | | |
| *TkWRKY34* | 3 | 2 | 2 | 1 | 2 | 1 | 1 | 2 | | | | 1 |

| | | | | | | | | | | | | |
|---|---|---|---|---|---|---|---|---|---|---|---|---|
| *TkWRKY35* | 2 | | | | | | | | 1 | 2 | | |
| *TkWRKY36* | | 1 | | | | | 6 | 3 | 2 | 1 | | 1 |
| *TkWRKY37* | | 1 | 1 | | 1 | | 4 | 2 | | | | |
| *TkWRKY38* | | | | | 1 | | 1 | 1 | | | | |
| *TkWRKY39* | | | 1 | | | | 1 | 6 | | 1 | | |
| *TkWRKY40* | | 1 | 1 | | 2 | | 4 | 2 | | | | |
| *TkWRKY41* | | | | | | | | 1 | | | 1 | |
| *TkWRKY42* | 1 | 1 | | | | 1 | 3 | 3 | | 2 | | |
| *TkWRKY43* | | | | | | | | | | | | |
| *TkWRKY44* | | | | | | | | | | | | |
| *TkWRKY45* | | | 1 | | | | 3 | 2 | | | | |
| *TkWRKY46* | | 2 | 1 | | 3 | | | 3 | | | | |
| *TkWRKY47* | 1 | 1 | 2 | | | | 1 | 1 | | 2 | | |
| *TkWRKY48* | 1 | | 1 | | | 1 | 1 | 1 | | | | |
| *TkWRKY49* | | 2 | 3 | | 2 | | 1 | 2 | | 2 | 1 | |
| *TkWRKY50* | 3 | | | | 2 | 1 | | | 1 | 1 | | |
| *TkWRKY51* | 1 | | | | 2 | | 1 | 3 | 2 | | | 1 |
| *TkWRKY52* | 1 | | 2 | | | 1 | 2 | 6 | 3 | 1 | | |
| *TkWRKY53* | | 2 | 2 | | 2 | | 2 | 1 | | 1 | | 1 |
| *TkWRKY54* | 3 | 1 | | | 2 | 1 | | 1 | | | | |
| *TkWRKY55* | 1 | | | | | | 2 | 1 | | | | |
| *TkWRKY56* | | | 1 | | 3 | | 2 | 1 | 1 | 2 | | |
| *TkWRKY57* | | 1 | | | | 1 | 2 | 1 | | 1 | | |
| *TkWRKY58* | 3 | | | | | 1 | 4 | 2 | | | | |
| *TkWRKY59* | | 1 | 1 | | 1 | | 3 | 1 | | 1 | | |
| *TkWRKY60* | 1 | | 2 | | | | 6 | 1 | 1 | | | |
| *TkWRKY61* | 1 | | | | | | 2 | 1 | | | | |
| *TkWRKY62* | | | | | 2 | | 1 | 5 | 2 | | | |
| *TkWRKY63* | 2 | | | | | | | 1 | | | | |
| *TkWRKY64* | | | 2 | | | | | 1 | 3 | | | |
| *TkWRKY65* | | | | | 1 | | 3 | 3 | | | | 1 |
| *TkWRKY66* | 1 | | | | 1 | 1 | 4 | 5 | | 1 | 1 | |
| *TkWRKY67* | | | | | | 2 | | 2 | | 1 | 1 | |
| *TkWRKY68* | 1 | | | | | 1 | 1 | 1 | 1 | | 1 | |

| | | | | | | | | | | | |
|---|---|---|---|---|---|---|---|---|---|---|---|
| *TkWRKY69* | 1 | | 3 | 2 | | 5 | | 2 | 2 | | 1 |
| *TkWRKY70* | | | | 1 | 1 | 1 | | 1 | | | |
| *TkWRKY71* | 1 | | 1 | 1 | | | 1 | | | | |
| *TkWRKY72* | | 1 | 1 | 4 | | 3 | 1 | 2 | | 1 | |

**Table S5** Transcriptome data sets of TKS WRKY genes.

| Gene name | FL_FPKM | LA_FPKM | PE_FPKM | SE_FPKM | ML_FPKM | MLR_FPKM |
|---|---|---|---|---|---|---|
| *TkWRKY1* | 17.1283 | 25.6379 | 17.33 | 15.5801 | 25.3646 | 11.5874 |
| *TkWRKY2* | 3.24154 | 0.027284 | 3.35413 | 3.50137 | 2.87067 | 0.155269 |
| *TkWRKY3* | 0.290672 | 0.882705 | 1.61459 | 1.01429 | 0.853402 | 15.7274 |
| *TkWRKY4* | 0.674436 | 17.235 | 0.210472 | 0.240001 | 0.145701 | 16.8435 |
| *TkWRKY5* | 0.176618 | 0 | 0.018132 | 0.019182 | 0 | 2.38568 |
| *TkWRKY6* | 11.2681 | 14.1192 | 5.79956 | 10.7103 | 7.30703 | 40.1169 |
| *TkWRKY7* | 19.2406 | 34.6835 | 15.1657 | 27.2162 | 19.4576 | 44.7096 |
| *TkWRKY8* | 7.63662 | 4.69867 | 12.842 | 1.07625 | 16.6598 | 40.1686 |
| *TkWRKY9* | 2.58751 | 0.23615 | 0.393898 | 0.448244 | 1.81974 | 22.4942 |
| *TkWRKY10* | 0.982251 | 0.377557 | 8.53716 | 0.338631 | 2.84284 | 9.1135 |
| *TkWRKY11* | 0.77463 | 0.499634 | 3.95982 | 0.255913 | 5.31735 | 0.293109 |
| *TkWRKY12* | 0.753264 | 0.243445 | 0.07404 | 1.22812 | 0.372937 | 15.9326 |
| *TkWRKY13* | 0.662385 | 0.050832 | 3.31544 | 0.314397 | 0.518081 | 0.578905 |
| *TkWRKY14* | 0.167701 | 0 | 0 | 0.387667 | 0.040846 | 0.206801 |
| *TkWRKY15* | 27.7187 | 27.5553 | 27.0901 | 1.43862 | 41.5982 | 14.2819 |
| *TkWRKY16* | 1.64948 | 0.283277 | 29.1895 | 0.228824 | 2.92124 | 0.356708 |
| *TkWRKY17* | 18.3457 | 2.49312 | 3.03255 | 31.167 | 36.0012 | 36.1623 |
| *TkWRKY18* | 3.93769 | 13.5105 | 1.40463 | 3.20872 | 3.90707 | 79.7124 |
| *TkWRKY19* | 5.65203 | 0.033123 | 3.15527 | 15.1585 | 4.14168 | 2.07942 |
| *TkWRKY20* | 0.618171 | 0 | 0.141997 | 0.239491 | 0.298461 | 0 |
| *TkWRKY21* | 0.099286 | 0.005387 | 0.006438 | 0 | 0.021098 | 1.53069 |
| *TkWRKY22* | 17.9256 | 15.9546 | 13.1867 | 12.4589 | 13.8824 | 69.3527 |
| *TkWRKY23* | 0.481367 | 9.23146 | 0.250646 | 0.279609 | 0.169042 | 12.2578 |
| *TkWRKY24* | 0.868471 | 0 | 0.018936 | 0.852567 | 17.2799 | 0 |
| *TkWRKY25* | 1.56653 | 4.7766 | 0.432362 | 6.43546 | 0.251806 | 1.45149 |
| *TkWRKY26* | 8.55675 | 0.784728 | 0.120417 | 0.906219 | 0.04739 | 1.70145 |
| *TkWRKY27* | 17.6033 | 11.7555 | 16.8449 | 1.35045 | 6.13082 | 13.7901 |
| *TkWRKY28* | 0.937802 | 158.341 | 0.49201 | 2.48425 | 2.33581 | 17.3944 |
| *TkWRKY29* | 0.76939 | 7.55464 | 0.46958 | 8.4627 | 0.213454 | 1.78805 |
| *TkWRKY30* | 0.312153 | 167.867 | 0.564192 | 0.037004 | 0.249615 | 12.7803 |
| *TkWRKY31* | 0.557373 | 7.61492 | 1.73079 | 0.179453 | 1.94941 | 11.1666 |
| *TkWRKY32* | 0 | 0.032836 | 0.020479 | 0 | 0 | 0 |
| *TkWRKY33* | 0.055974 | 0.15262 | 0.093552 | 0.029676 | 0.298472 | 1.50641 |
| *TkWRKY34* | 0.038962 | 0.019872 | 0.013105 | 0.069275 | 0.307904 | 0.2619 |
| *TkWRKY35* | 12.3045 | 10.2264 | 11.2701 | 12.6406 | 16.9612 | 12.0884 |

| | | | | | | |
|---|---|---|---|---|---|---|
| *TkWRKY36* | 13.5665 | 0.333059 | 5.30896 | 10.8717 | 6.51826 | 1.43157 |
| *TkWRKY37* | 0.076222 | 1.59566 | 0.120928 | 3.89945 | 1.42499 | 69.4778 |
| *TkWRKY38* | 4.61937 | 121.474 | 8.25753 | 54.3085 | 4.75565 | 158.479 |
| *TkWRKY39* | 0 | 0.095286 | 0.01868 | 0 | 0 | 0.358859 |
| *TkWRKY40* | 6.62515 | 0.078664 | 3.75807 | 15.1289 | 3.83079 | 2.06749 |
| *TkWRKY41* | 0 | 0.029078 | 0 | 0 | 0 | 1.1453 |
| *TkWRKY42* | 0.70748 | 0.520264 | 1.03831 | 1.46064 | 0.098481 | 0.230252 |
| *TkWRKY43* | 0.654554 | 0.199247 | 7.75582 | 0.125559 | 1.72701 | 5.77248 |
| *TkWRKY44* | 3.32635 | 0 | 2.74791 | 4.03579 | 2.4481 | 0.045164 |
| *TkWRKY45* | 0.318305 | 0 | 0.144888 | 0.045927 | 3.78566 | 0 |
| *TkWRKY46* | 6.4446 | 3.57092 | 6.8158 | 8.11957 | 6.76002 | 4.35661 |
| *TkWRKY47* | 0.583452 | 0.133745 | 4.05963 | 0.171655 | 7.19755 | 0.409455 |
| *TkWRKY48* | 32.3803 | 32.8205 | 33.2324 | 36.6129 | 34.2102 | 61.8198 |
| *TkWRKY49* | 0.665393 | 0.941672 | 2.39687 | 1.50345 | 0.460404 | 20.9335 |
| *TkWRKY50* | 0.803838 | 0.523677 | 1.18741 | 0.029262 | 5.55777 | 16.1493 |
| *TkWRKY51* | 0.457558 | 0.792458 | 0.198847 | 0.281261 | 0.344002 | 1.77072 |
| *TkWRKY52* | 5.95971 | 4.58298 | 2.46348 | 0.774355 | 1.48424 | 37.5001 |
| *TkWRKY53* | 0.050831 | 0 | 0 | 0.189185 | 0.012611 | 0.094395 |
| *TkWRKY54* | 0.034685 | 0 | 0.019334 | 0.112031 | 0 | 0.071014 |
| *TkWRKY55* | 0.029312 | 0 | 0 | 0.511567 | 0 | 0.616797 |
| *TkWRKY56* | 38.8896 | 29.2058 | 22.1152 | 28.1781 | 34.7593 | 96.0577 |
| *TkWRKY57* | 0.714416 | 0.070814 | 2.24947 | 0.686409 | 1.83242 | 1.57069 |
| *TkWRKY58* | 5.8476 | 6.56318 | 2.15682 | 0.71002 | 6.89375 | 2.9358 |
| *TkWRKY59* | 0.099216 | 0.255925 | 0.009966 | 0.053477 | 0.019514 | 28.9537 |
| *TkWRKY60* | 6.75774 | 18.1893 | 12.2561 | 7.78365 | 5.89771 | 31.8113 |
| *TkWRKY61* | 0 | 0 | 0 | 0 | 0 | 0 |
| *TkWRKY62* | 0.53172 | 2.43793 | 0.295783 | 0.329318 | 0.610856 | 52.1431 |
| *TkWRKY63* | 7.66047 | 39.573 | 6.8866 | 27.1465 | 23.6996 | 158.872 |
| *TkWRKY64* | 28.836 | 78.2093 | 41.6308 | 91.3014 | 59.1813 | 172.513 |
| *TkWRKY65* | 0.331709 | 4.50554 | 0.197498 | 4.77706 | 0.185672 | 108.335 |
| *TkWRKY66* | 1.94928 | 39.2037 | 5.17355 | 1.3273 | 5.81662 | 32.5031 |
| *TkWRKY67* | 16.4511 | 18.8646 | 13.4108 | 16.0638 | 12.9283 | 12.9691 |
| *TkWRKY68* | 0.364387 | 0.09232 | 0.930194 | 0.38696 | 1.59925 | 2.61198 |
| *TkWRKY69* | 0.07098 | 0.020933 | 0 | 4.07984 | 0.1117 | 1.62356 |
| *TkWRKY70* | 1.2315 | 2.78109 | 0.250086 | 0.261498 | 2.15892 | 37.4675 |
| *TkWRKY71* | 7.6969 | 22.3617 | 7.98214 | 2.93636 | 36.0474 | 393.654 |
| *TkWRKY72* | 0.60969 | 0.946208 | 0.346008 | 0 | 3.56286 | 2.17346 |

| Gene name | MMR_FPKM | MS_FPKM | YL_FPKM | YLR_FPKM | YMR_FPKM | YS_FPKM |
|---|---|---|---|---|---|---|
| *TkWRKY1* | 10.6372 | 14.9978 | 25.2227 | 18.62 | 12.7659 | 21.4787 |
| *TkWRKY2* | 0.066427 | 0.120224 | 1.07893 | 0.693342 | 0.406004 | 0.737356 |
| *TkWRKY3* | 6.89491 | 6.83025 | 0.574483 | 8.29445 | 10.7744 | 4.26818 |
| *TkWRKY4* | 6.9991 | 3.96322 | 0.06021 | 6.07575 | 7.96165 | 5.86123 |
| *TkWRKY5* | 0.254441 | 0.208882 | 0 | 3.72902 | 1.63246 | 0.773169 |
| *TkWRKY6* | 14.8044 | 16.7538 | 6.66828 | 32.5981 | 44.0507 | 38.6722 |
| *TkWRKY7* | 47.6204 | 34.075 | 17.3202 | 24.152 | 55.8573 | 62.6127 |
| *TkWRKY8* | 35.6866 | 19.1475 | 16.4979 | 16.3345 | 16.6439 | 10.4284 |
| *TkWRKY9* | 8.60452 | 4.33695 | 7.48983 | 27.4336 | 12.6894 | 1.99077 |
| *TkWRKY10* | 2.23002 | 9.63399 | 7.20687 | 3.71989 | 2.42788 | 0.94348 |
| *TkWRKY11* | 0.263059 | 0.274211 | 6.75598 | 0.231451 | 0.337475 | 0.8303 |
| *TkWRKY12* | 1.66602 | 1.66372 | 0.019757 | 53.0382 | 18.3924 | 20.3207 |
| *TkWRKY13* | 0.272422 | 0.062245 | 0.360915 | 3.25498 | 1.78245 | 0.296409 |
| *TkWRKY14* | 0.02054 | 0.04468 | 0 | 1.86608 | 0.160823 | 0.13938 |
| *TkWRKY15* | 5.37852 | 28.113 | 13.4419 | 18.8407 | 17.3387 | 24.2895 |
| *TkWRKY16* | 0.512625 | 5.91676 | 4.5467 | 0.316413 | 1.05243 | 12.4578 |
| *TkWRKY17* | 11.6684 | 10.2765 | 5.81566 | 67.2961 | 40.2996 | 40.8831 |
| *TkWRKY18* | 54.0617 | 44.4635 | 2.02395 | 141.5 | 215.33 | 205.351 |
| *TkWRKY19* | 0.970274 | 0.476192 | 2.04212 | 2.91195 | 1.68541 | 1.32555 |
| *TkWRKY20* | 0 | 0 | 0.177329 | 0.790501 | 0 | 0 |
| *TkWRKY21* | 0.075728 | 0.145294 | 0 | 6.17012 | 1.62753 | 0.259618 |
| *TkWRKY22* | 46.7375 | 45.0898 | 9.93295 | 70.9356 | 55.8403 | 29.599 |
| *TkWRKY23* | 5.93927 | 3.04379 | 0.04073 | 7.72849 | 7.66959 | 5.79663 |
| *TkWRKY24* | 0 | 0.018126 | 6.41568 | 0 | 0 | 0 |
| *TkWRKY25* | 0.476949 | 1.20318 | 0.191856 | 2.29982 | 0.789151 | 0.757314 |
| *TkWRKY26* | 2.27447 | 3.49038 | 0.019757 | 1.17086 | 1.21534 | 2.21932 |
| *TkWRKY27* | 8.39814 | 51.1421 | 10.5045 | 9.24521 | 16.3927 | 11.2471 |
| *TkWRKY28* | 10.5403 | 14.6341 | 0.736686 | 14.5612 | 10.9437 | 15.0842 |
| *TkWRKY29* | 0.643699 | 0.99796 | 0.120515 | 2.12534 | 1.24355 | 0.639702 |
| *TkWRKY30* | 8.96497 | 5.62071 | 0.097526 | 9.87474 | 12.8396 | 9.7183 |
| *TkWRKY31* | 9.40394 | 9.63309 | 9.07138 | 13.1247 | 11.7991 | 6.99318 |
| *TkWRKY32* | 0.02285 | 0.084687 | 0 | 0.278015 | 0.02187 | 0.020901 |
| *TkWRKY33* | 0.388569 | 0.46225 | 0.450988 | 1.55745 | 0.220039 | 0.028657 |
| *TkWRKY34* | 0.013116 | 0.074795 | 0 | 3.96272 | 0.414623 | 0.02744 |
| *TkWRKY35* | 10.2934 | 12.5586 | 15.8768 | 16.674 | 14.8438 | 14.9549 |
| *TkWRKY36* | 0.822661 | 1.27941 | 9.43088 | 1.5932 | 2.04882 | 2.97459 |

| | | | | | | |
|---|---|---|---|---|---|---|
| *TkWRKY37* | 38.5622 | 20.5905 | 0.305816 | 40.607 | 33.7904 | 6.19077 |
| *TkWRKY38* | 197.048 | 120.13 | 2.3103 | 43.1324 | 135.046 | 85.5722 |
| *TkWRKY39* | 0.233369 | 2.03803 | 0 | 0.017471 | 0.0854 | 0.259363 |
| *TkWRKY40* | 0.60542 | 0.370394 | 2.20275 | 3.31204 | 1.57794 | 1.18553 |
| *TkWRKY41* | 0.101093 | 0 | 0 | 5.76157 | 1.60302 | 0 |
| *TkWRKY42* | 0.042224 | 0.340283 | 0.435972 | 0.371221 | 0.151845 | 0.717741 |
| *TkWRKY43* | 1.70341 | 8.20773 | 2.20199 | 2.6753 | 2.21623 | 1.08536 |
| *TkWRKY44* | 0.084009 | 0.180658 | 1.00123 | 0.216328 | 0.030397 | 0.309564 |
| *TkWRKY45* | 0 | 0.076446 | 3.47733 | 0 | 0 | 0.035606 |
| *TkWRKY46* | 2.89292 | 3.8065 | 7.22938 | 4.41536 | 4.45116 | 3.99509 |
| *TkWRKY47* | 0.230898 | 0.34438 | 6.53236 | 0.218251 | 0.289326 | 0.893334 |
| *TkWRKY48* | 77.9903 | 83.3277 | 25.7757 | 35.1675 | 42.793 | 29.4103 |
| *TkWRKY49* | 10.9304 | 10.3557 | 1.4878 | 12.9326 | 11.7923 | 6.88214 |
| *TkWRKY50* | 6.53274 | 4.02417 | 0.532448 | 11.2082 | 28.1438 | 32.5843 |
| *TkWRKY51* | 0.711902 | 0.986585 | 0.238967 | 2.14765 | 1.84331 | 1.08823 |
| *TkWRKY52* | 6.69967 | 95.4851 | 1.36951 | 33.5655 | 22.0967 | 14.0501 |
| *TkWRKY53* | 0 | 0.032369 | 0.013525 | 1.6923 | 0.149391 | 0.173181 |
| *TkWRKY54* | 0 | 0.039217 | 0.046704 | 0.774748 | 0.057755 | 0.093086 |
| *TkWRKY55* | 0.089662 | 0 | 0 | 1.19065 | 0.513325 | 0.159201 |
| *TkWRKY56* | 40.9145 | 56.0356 | 21.1038 | 99.0179 | 98.0083 | 77.2679 |
| *TkWRKY57* | 0.765006 | 5.57521 | 2.1429 | 2.09201 | 3.16651 | 9.4029 |
| *TkWRKY58* | 2.1868 | 19.333 | 4.26992 | 4.23867 | 3.83539 | 8.32199 |
| *TkWRKY59* | 6.82959 | 10.6442 | 0.012767 | 10.0791 | 10.1933 | 7.80811 |
| *TkWRKY60* | 22.102 | 69.465 | 17.0546 | 17.1535 | 18.2569 | 27.7554 |
| *TkWRKY61* | 0 | 0 | 0 | 0.212326 | 0.039873 | 0 |
| *TkWRKY62* | 16.4222 | 6.3412 | 0.215222 | 70.6201 | 121.064 | 101.899 |
| *TkWRKY63* | 91.0756 | 120.887 | 9.29633 | 140.218 | 176.012 | 153.679 |
| *TkWRKY64* | 226.603 | 91.2839 | 50.0428 | 112.632 | 182.601 | 98.992 |
| *TkWRKY65* | 48.6155 | 14.9431 | 0.189324 | 53.1711 | 69.256 | 26.6543 |
| *TkWRKY66* | 29.9317 | 24.7035 | 1.80759 | 27.0487 | 31.3961 | 24.7135 |
| *TkWRKY67* | 13.7576 | 19.6653 | 13.5606 | 10.1792 | 13.6959 | 14.8603 |
| *TkWRKY68* | 0.689443 | 0.143333 | 0.703647 | 3.55371 | 1.27099 | 0.526074 |
| *TkWRKY69* | 0.35107 | 0.274724 | 0.030515 | 2.9786 | 0.553112 | 0.609663 |
| *TkWRKY70* | 8.79878 | 39.308 | 0.244617 | 19.6802 | 50.2113 | 50.8552 |
| *TkWRKY71* | 206.132 | 208.295 | 8.04607 | 393.543 | 373.731 | 322.925 |
| *TkWRKY72* | 1.17687 | 0.452387 | 0.0185 | 8.84855 | 5.82644 | 5.3123 |

**Table S6** Primers for Real-time RT-PCR.

| Primer name | Primer Sequence | Primer name | Primer Sequence |
|---|---|---|---|
| Q*TkWRKY4*-F | CAGCTACAGATTCATCCTCCCC | Q*TkWRKY4*-R | TTATTTGCAGCCTCGTTTGACG |
| Q*TkWRKY6*-F | ATCGGATGTTCCAGTTGACCAA | Q*TkWRKY6*-R | TAGAAGTAGAAGGCGCGTTTGT |
| Q*TkWRKY10*-F | TCCTGTTGGCCTGAGAGTTTAC | Q*TkWRKY10*-R | AAGATCTGCTTCACCACATCGT |
| Q*TkWRKY13*-F | GGTTAAGTCGATACGTGAGCCA | Q*TkWRKY13*-R | CCTGGGATTAGGGTTGCTTCTT |
| Q*TkWRKY15*-F | AACTCCAAATCCCCAAGGTGTT | Q*TkWRKY15*-R | GCAAGTGTGGTGGCCAAAATAT |
| Q*TkWRKY18*-F | AATGGTGGTCAACATCCGTACA | Q*TkWRKY18*-R | CTTCGGGTTCTTCCTTAGCACT |
| Q*TkWRKY21*-F | GGCTAAGACTCCATTTGGACCA | Q*TkWRKY21*-R | TCTTTATGGTGGATTGTGGGGG |
| Q*TkWRKY23*-F | CAGCTACAGATTCATCCTCCCC | Q*TkWRKY23*-R | TTATTTGCAGCCTCGTTTGACG |
| Q*TkWRKY27*-F | GACTCAGGCGAAAGTGAGAAGA | Q*TkWRKY27*-R | TAGCACCTTGGAAATGTAGCGT |
| Q*TkWRKY28*-F | ATGGTAAGGAGGTGATCATGGC | Q*TkWRKY28*-R | TCTCCATCTATAGCCGTCGTCA |
| Q*TkWRKY38*-F | ACTGGAAGAATCTCAGCAGCAA | Q*TkWRKY38*-R | TGGAACTGAGATGGGTGTTCTG |
| Q*TkWRKY39*-F | CAGCTAGTGCAACATCAACACC | Q*TkWRKY39*-R | TGTGTTCTTCTCCGAGTTCGTT |
| Q*TkWRKY56*-F | ACCAGTAATCAACCGGTTGTGT | Q*TkWRKY56*-R | GGTTTACCACCAGCAGATAGCT |
| Q*TkWRKY71*-F | TAACTCCAAGAAACGGAAGCCA | Q*TkWRKY71*-R | AGGTGTTGTTCTTGTCTGGGTT |
| *GAPDH*-F | AGTTGGTTTCGTGGTATGAC | *GAPDH*-R | ACATGTCAGTGAACAGGTAGAC |
| *β-actin*-F | GGAAGGATCTTTATGGGAAC | *β-actin*-R | CAGACACTATACTTCCTCTCAGG |
