# Supplementary material for: Identification of the WRKY Gene Family and Characterization of Stress-Responsive Genes in Taraxacum kok-saghyz Rodin
Source: Int J Mol Sci. 2022 Sep 7;23(18):10270. doi: 10.3390/ijms231810270 (PMC9499643; doi:10.3390/ijms231810270)
Supplement: Supplementary file 1 [file ijms-23-10270-s001.zip › Table S2 The 20 motifs in TKS WRKY proteins.pdf]

Table S2 The 20 motifs in *Taraxacum kok-saghyz* Rodin WRKY proteins.

Motif number	Motif Sequence	Motif Logo	Width
Motif 1	DGYRWRKYGQKVVKG	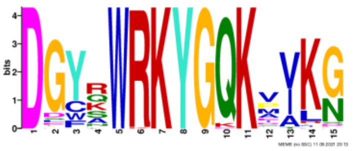	15
Motif 2	SPHPRSYYRCTSAGC	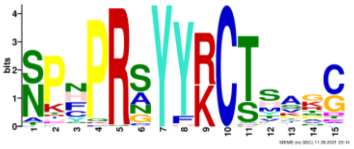	15
Motif 3	DPSIVITTYEGKHNH	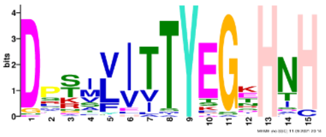	15
Motif 4	DDGYNWRKYGQKQVKGS<br>EYPRSYYKCTHPNCPVKK	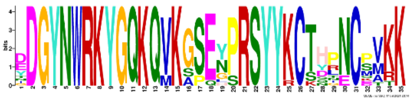	35
Motif 5	KKIREPRVAVQTRSEVDILD	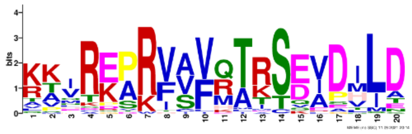	20
Motif 6	PVRKQVZRASD	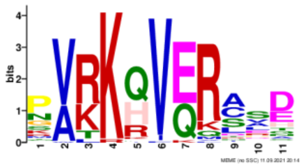	11
Motif 7	GQITEIVYKGNHNHPKPQPTK	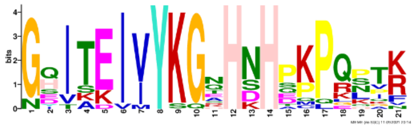	21
Motif 8	PLPPAATAMASTTSAAASMLL	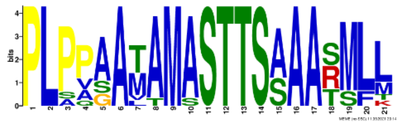	21
Motif 9	VQAELERMNEENQRLREMLDQV<br>MKBYNALQMHLKTVMQQKQQEE	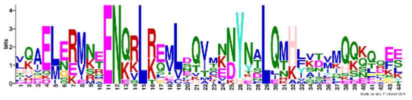	44
Motif 10	ALAETTTAJTADPNFTAALAAAISS<br>IIGG	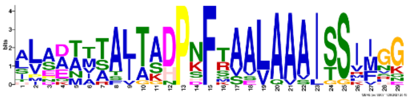	29
Motif 11	SSGRCHCSKKRKHRMKR<br>VVRVPAISMKLADIPP	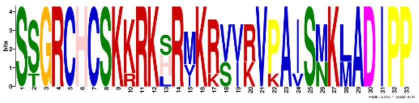	33

Motif 12	GJSPSNFLESPFLLTNSSIAPSPTSGSFP	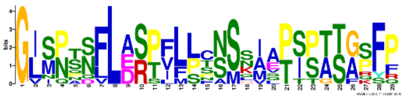	29
Motif 13	GFGTGISPGPMTLVSNFFSDHYPDADLRSFSQLLAGVIPSPAAEYPPPHC	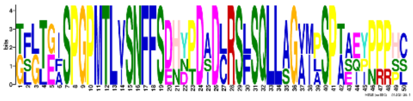	50
Motif 14	KKKLIEELTQGQELAKKLKRLLRRPQSSE	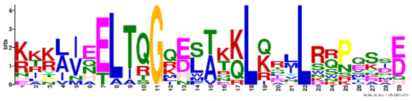	29
Motif 15	GGGGGGGFFDTPQGYLDMLAFQDYGGASLFDLLQQPTPPNLVVEQLQIH	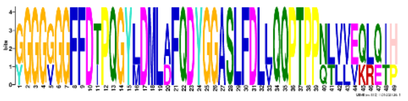	49
Motif 16	LVDEILGSFKKALSLLNST	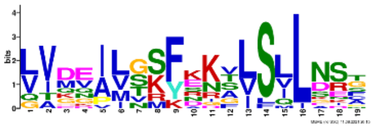	19
Motif 17	VTPVKERRGCYKRRKTSDSRVKIAPTIE	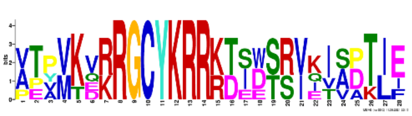	28
Motif 18	PFMDHGLAPPSLVEDEEIPTSSADGHDEE	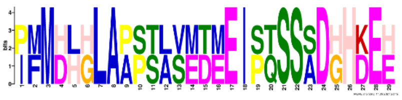	29
Motif 19	PTTIQFPLNLNCNHDHQEKQINEMDFFSDKKHDEDRSRLPV	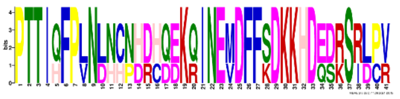	41
Motif 20	PTRIPYFNYSLNPTTYAPAMDHGGLLQDVLAYHRQGTPADH	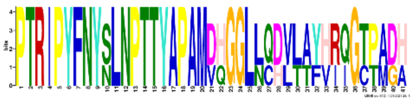	41

Note: In Motif Logo, the larger the letter, the more conserved the amino acid.
