# Supplementary material for: Identification of the WRKY Gene Family and Characterization of Stress-Responsive Genes in Taraxacum kok-saghyz Rodin
Source: Int J Mol Sci. 2022 Sep 7;23(18):10270. doi: 10.3390/ijms231810270 (PMC9499643; doi:10.3390/ijms231810270)
Supplement: Supplementary file 1 [file ijms-23-10270-s001.zip › Table S3 The 20 motifs in nine species WRKY proteins.pdf]

Table S3 The 20 motifs in nine species (rice, maize, *Brachypodium distachyon*, pineapple, *Arabidopsis*, grapes, peaches, poplars and *Taraxacum kok-saghyz* Rodin) WRKY proteins.

Motif number	Motif Sequence	Motif Logo	Width
Motif 1	PIDDGYRWRKYGQKHILGAKHPRSYYRC	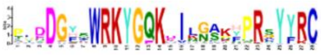	28
Motif 2	DPHLFLYTYRGLHTC	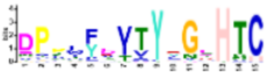	15
Motif 3	DQGCTATKQVQRSDD	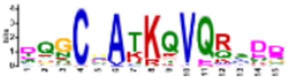	15
Motif 4	LYLELYRGKELAKQLETLLRP	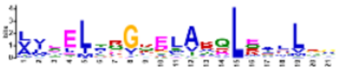	21
Motif 5	ERVELLVQKILSSFERALSILN	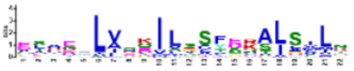	22
Motif 6	KKRKTLPRWTEQVRV	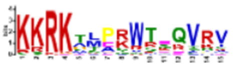	15
Motif 7	VKDRRGCYKRRKTSQSWTTV	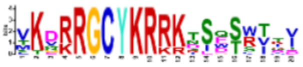	20
Motif 8	ESDLTEIISAATSVTNSPIVD	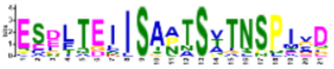	21
Motif 9	PSFISPATSESNYFSVSPCQM	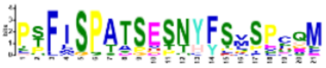	21
Motif 10	AQEHNPQAQGLLQSLQAGLTVKTEGLD	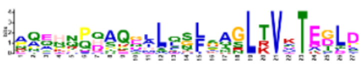	27
Motif 11	LDDGYRWRKYGQKVVKGNPRSYYKCT	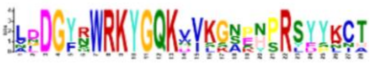	28
Motif 12	YVDLADAMFNSGSSSTN	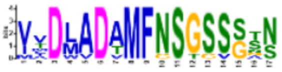	17
Motif 13	VEFDPNFPFDNPGFF	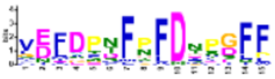	15

Motif 14	SSSQRPRRRKDEGERRTYRVPAQRTGNTEI	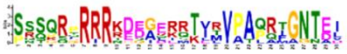	30
Motif 15	KAFAVHDDTYGSEMIKFDQVVSESVMPQLATI DEQAITMED	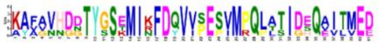	41
Motif 16	HIMNQECDINDYLVDDDPFWASQFPPF	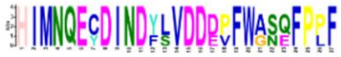	27
Motif 17	SPNGSLSTHVASYPVFEYAEWRGDADLQEVVS	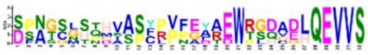	32
Motif 18	LPSSSIIIPFFSTPCTSNSNQPQKNNYFTESVISNL VMTGF	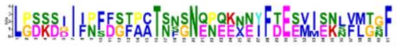	41
Motif 19	SPSSGNGSPRSEDSD	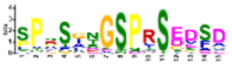	15
Motif 20	SGLDHGDVISSVNYSSTTSTHSYDIDNM	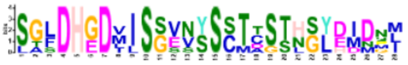	28

Note: In Motif Logo, the larger the letter, the more conserved the amino acid.
