# Supplementary material for: Identification of the WRKY Gene Family and Characterization of Stress-Responsive Genes in Taraxacum kok-saghyz Rodin
Source: Int J Mol Sci. 2022 Sep 7;23(18):10270. doi: 10.3390/ijms231810270 (PMC9499643; doi:10.3390/ijms231810270)
Supplement: Supplementary file 1 [file ijms-23-10270-s001.zip › Table S4 Statistics of cis-acting elements of TkWRKY genes.pdf]

Table S4 Statistics of *cis*-acting elements of TkWRKY genes.

Gene name	WUN-motif	MBS	LTR	MBSI	W-box	TC-rich repeats	CGTCA-motif	ABRE	TCA-element	TGA-element	GARE-motif	circadian
TkWRKY1	1	1			1	1	3	1				
TkWRKY2	1	1	1		1		2	1		1		
TkWRKY3		1	2		1		2	1		2	1	
TkWRKY4			1		2	1	3	2				1
TkWRKY5	1	1			2	1	1	2	3			
TkWRKY6			5			1	3	1				1
TkWRKY7	1				2	1	3			1		
TkWRKY8		1			1		4	1		2		
TkWRKY9			1		1		1	1		2		1
TkWRKY10		1	1		3		1	5				
TkWRKY11	1	1	2					1	2	2		
TkWRKY12	1				1		5	5	2			
TkWRKY13	1	1	1		1		2	1	1	2		
TkWRKY14		1	3		1	1	3	3		1		
TkWRKY15	1	1	2				4	3	2	1		
TkWRKY16	1	1					1	3	1			
TkWRKY17	1	1	1		1	1	2	2	1			
TkWRKY18		2	2		2		1		1	1	1	1
TkWRKY19		2	1		2		3	2				
TkWRKY20					2	1		1				
TkWRKY21	4	1	1						1		1	
TkWRKY22	1	1	1				6	2		1		1
TkWRKY23					2	2	3	2				
TkWRKY24	4				3		3	1	2			
TkWRKY25							5	2				
TkWRKY26	2				1	1	1	7				
TkWRKY27	1	4	1				3	1				
TkWRKY28	1				5			2	1			
TkWRKY29			1	1		1	2	4				
TkWRKY30		1			2		3	3				1
TkWRKY31	1				1			4				

TkWRKY32	1		1		1	2	3	1				
TkWRKY33		1	2				2	1		1		
TkWRKY34	3	2	2	1	2	1	1	2				1
TkWRKY35	2								1	2		
TkWRKY36		1					6	3	2	1		1
TkWRKY37		1	1		1		4	2				
TkWRKY38					1		1	1				
TkWRKY39			1				1	6		1		
TkWRKY40		1	1		2		4	2				
TkWRKY41								1			1	
TkWRKY42	1	1				1	3	3		2		
TkWRKY43												
TkWRKY44												
TkWRKY45			1				3	2				
TkWRKY46		2	1		3			3				
TkWRKY47	1	1	2				1	1		2		
TkWRKY48	1		1			1	1	1				
TkWRKY49		2	3		2		1	2		2	1	
TkWRKY50	3				2	1			1	1		
TkWRKY51	1				2		1	3	2			1
TkWRKY52	1		2			1	2	6	3	1		
TkWRKY53		2	2		2		2	1		1		1
TkWRKY54	3	1			2	1		1				
TkWRKY55	1						2	1				
TkWRKY56			1		3		2	1	1	2		
TkWRKY57		1				1	2	1		1		
TkWRKY58	3					1	4	2				
TkWRKY59		1	1		1		3	1		1		
TkWRKY60	1		2				6	1	1			
TkWRKY61	1						2	1				
TkWRKY62					2		1	5	2			
TkWRKY63	2							1				
TkWRKY64			2					1	3			
TkWRKY65					1		3	3				1

TkWRKY66	1			1	1	4	5		1	1	
TkWRKY67					2		2		1	1	
TkWRKY68	1				1	1	1	1		1	
TkWRKY69	1		3	2		5		2	2		1
TkWRKY70				1	1	1		1			
TkWRKY71	1		1	1			1				
TkWRKY72		1	1	4		3	1	2		1	
