# Supplementary material for: Identification of the WRKY Gene Family and Characterization of Stress-Responsive Genes in Taraxacum kok-saghyz Rodin
Source: Int J Mol Sci. 2022 Sep 7;23(18):10270. doi: 10.3390/ijms231810270 (PMC9499643; doi:10.3390/ijms231810270)
Supplement: Supplementary file 1 [file ijms-23-10270-s001.zip › Table S5 Transcriptome data sets of TKS WRKY genes.pdf]

Table S5 Transcriptome data sets of TKS WRKY genes.

Gene name	FL_FPKM	LA_FPKM	PE_FPKM	SE_FPKM	ML_FPKM	MLR_FPKM
TkWRKY1	17.1283	25.6379	17.33	15.5801	25.3646	11.5874
TkWRKY2	3.24154	0.027284	3.35413	3.50137	2.87067	0.155269
TkWRKY3	0.290672	0.882705	1.61459	1.01429	0.853402	15.7274
TkWRKY4	0.674436	17.235	0.210472	0.240001	0.145701	16.8435
TkWRKY5	0.176618	0	0.018132	0.019182	0	2.38568
TkWRKY6	11.2681	14.1192	5.79956	10.7103	7.30703	40.1169
TkWRKY7	19.2406	34.6835	15.1657	27.2162	19.4576	44.7096
TkWRKY8	7.63662	4.69867	12.842	1.07625	16.6598	40.1686
TkWRKY9	2.58751	0.23615	0.393898	0.448244	1.81974	22.4942
TkWRKY10	0.982251	0.377557	8.53716	0.338631	2.84284	9.1135
TkWRKY11	0.77463	0.499634	3.95982	0.255913	5.31735	0.293109
TkWRKY12	0.753264	0.243445	0.07404	1.22812	0.372937	15.9326
TkWRKY13	0.662385	0.050832	3.31544	0.314397	0.518081	0.578905
TkWRKY14	0.167701	0	0	0.387667	0.040846	0.206801
TkWRKY15	27.7187	27.5553	27.0901	1.43862	41.5982	14.2819
TkWRKY16	1.64948	0.283277	29.1895	0.228824	2.92124	0.356708
TkWRKY17	18.3457	2.49312	3.03255	31.167	36.0012	36.1623
TkWRKY18	3.93769	13.5105	1.40463	3.20872	3.90707	79.7124
TkWRKY19	5.65203	0.033123	3.15527	15.1585	4.14168	2.07942
TkWRKY20	0.618171	0	0.141997	0.239491	0.298461	0
TkWRKY21	0.099286	0.005387	0.006438	0	0.021098	1.53069
TkWRKY22	17.9256	15.9546	13.1867	12.4589	13.8824	69.3527
TkWRKY23	0.481367	9.23146	0.250646	0.279609	0.169042	12.2578
TkWRKY24	0.868471	0	0.018936	0.852567	17.2799	0
TkWRKY25	1.56653	4.7766	0.432362	6.43546	0.251806	1.45149
TkWRKY26	8.55675	0.784728	0.120417	0.906219	0.04739	1.70145
TkWRKY27	17.6033	11.7555	16.8449	1.35045	6.13082	13.7901
TkWRKY28	0.937802	158.341	0.49201	2.48425	2.33581	17.3944
TkWRKY29	0.76939	7.55464	0.46958	8.4627	0.213454	1.78805
TkWRKY30	0.312153	167.867	0.564192	0.037004	0.249615	12.7803
TkWRKY31	0.557373	7.61492	1.73079	0.179453	1.94941	11.1666
TkWRKY32	0	0.032836	0.020479	0	0	0
TkWRKY33	0.055974	0.15262	0.093552	0.029676	0.298472	1.50641
TkWRKY34	0.038962	0.019872	0.013105	0.069275	0.307904	0.2619
TkWRKY35	12.3045	10.2264	11.2701	12.6406	16.9612	12.0884
TkWRKY36	13.5665	0.333059	5.30896	10.8717	6.51826	1.43157
TkWRKY37	0.076222	1.59566	0.120928	3.89945	1.42499	69.4778
TkWRKY38	4.61937	121.474	8.25753	54.3085	4.75565	158.479
TkWRKY39	0	0.095286	0.01868	0	0	0.358859
TkWRKY40	6.62515	0.078664	3.75807	15.1289	3.83079	2.06749
TkWRKY41	0	0.029078	0	0	0	1.1453
TkWRKY42	0.70748	0.520264	1.03831	1.46064	0.098481	0.230252

TkWRKY43	0.654554	0.199247	7.75582	0.125559	1.72701	5.77248
TkWRKY44	3.32635	0	2.74791	4.03579	2.4481	0.045164
TkWRKY45	0.318305	0	0.144888	0.045927	3.78566	0
TkWRKY46	6.4446	3.57092	6.8158	8.11957	6.76002	4.35661
TkWRKY47	0.583452	0.133745	4.05963	0.171655	7.19755	0.409455
TkWRKY48	32.3803	32.8205	33.2324	36.6129	34.2102	61.8198
TkWRKY49	0.665393	0.941672	2.39687	1.50345	0.460404	20.9335
TkWRKY50	0.803838	0.523677	1.18741	0.029262	5.55777	16.1493
TkWRKY51	0.457558	0.792458	0.198847	0.281261	0.344002	1.77072
TkWRKY52	5.95971	4.58298	2.46348	0.774355	1.48424	37.5001
TkWRKY53	0.050831	0	0	0.189185	0.012611	0.094395
TkWRKY54	0.034685	0	0.019334	0.112031	0	0.071014
TkWRKY55	0.029312	0	0	0.511567	0	0.616797
TkWRKY56	38.8896	29.2058	22.1152	28.1781	34.7593	96.0577
TkWRKY57	0.714416	0.070814	2.24947	0.686409	1.83242	1.57069
TkWRKY58	5.8476	6.56318	2.15682	0.71002	6.89375	2.9358
TkWRKY59	0.099216	0.255925	0.009966	0.053477	0.019514	28.9537
TkWRKY60	6.75774	18.1893	12.2561	7.78365	5.89771	31.8113
TkWRKY61	0	0	0	0	0	0
TkWRKY62	0.53172	2.43793	0.295783	0.329318	0.610856	52.1431
TkWRKY63	7.66047	39.573	6.8866	27.1465	23.6996	158.872
TkWRKY64	28.836	78.2093	41.6308	91.3014	59.1813	172.513
TkWRKY65	0.331709	4.50554	0.197498	4.77706	0.185672	108.335
TkWRKY66	1.94928	39.2037	5.17355	1.3273	5.81662	32.5031
TkWRKY67	16.4511	18.8646	13.4108	16.0638	12.9283	12.9691
TkWRKY68	0.364387	0.09232	0.930194	0.38696	1.59925	2.61198
TkWRKY69	0.07098	0.020933	0	4.07984	0.1117	1.62356
TkWRKY70	1.2315	2.78109	0.250086	0.261498	2.15892	37.4675
TkWRKY71	7.6969	22.3617	7.98214	2.93636	36.0474	393.654
TkWRKY72	0.60969	0.946208	0.346008	0	3.56286	2.17346

Gene name	MMR_FPKM	MS_FPKM	YL_FPKM	YLR_FPKM	YMR_FPKM	YS_FPKM
TkWRKY1	10.6372	14.9978	25.2227	18.62	12.7659	21.4787
TkWRKY2	0.066427	0.120224	1.07893	0.693342	0.406004	0.737356
TkWRKY3	6.89491	6.83025	0.574483	8.29445	10.7744	4.26818
TkWRKY4	6.9991	3.96322	0.06021	6.07575	7.96165	5.86123
TkWRKY5	0.254441	0.208882	0	3.72902	1.63246	0.773169
TkWRKY6	14.8044	16.7538	6.66828	32.5981	44.0507	38.6722
TkWRKY7	47.6204	34.075	17.3202	24.152	55.8573	62.6127
TkWRKY8	35.6866	19.1475	16.4979	16.3345	16.6439	10.4284
TkWRKY9	8.60452	4.33695	7.48983	27.4336	12.6894	1.99077
TkWRKY10	2.23002	9.63399	7.20687	3.71989	2.42788	0.94348
TkWRKY11	0.263059	0.274211	6.75598	0.231451	0.337475	0.8303
TkWRKY12	1.66602	1.66372	0.019757	53.0382	18.3924	20.3207
TkWRKY13	0.272422	0.062245	0.360915	3.25498	1.78245	0.296409
TkWRKY14	0.02054	0.04468	0	1.86608	0.160823	0.13938
TkWRKY15	5.37852	28.113	13.4419	18.8407	17.3387	24.2895
TkWRKY16	0.512625	5.91676	4.5467	0.316413	1.05243	12.4578
TkWRKY17	11.6684	10.2765	5.81566	67.2961	40.2996	40.8831
TkWRKY18	54.0617	44.4635	2.02395	141.5	215.33	205.351
TkWRKY19	0.970274	0.476192	2.04212	2.91195	1.68541	1.32555
TkWRKY20	0	0	0.177329	0.790501	0	0
TkWRKY21	0.075728	0.145294	0	6.17012	1.62753	0.259618
TkWRKY22	46.7375	45.0898	9.93295	70.9356	55.8403	29.599
TkWRKY23	5.93927	3.04379	0.04073	7.72849	7.66959	5.79663
TkWRKY24	0	0.018126	6.41568	0	0	0
TkWRKY25	0.476949	1.20318	0.191856	2.29982	0.789151	0.757314
TkWRKY26	2.27447	3.49038	0.019757	1.17086	1.21534	2.21932
TkWRKY27	8.39814	51.1421	10.5045	9.24521	16.3927	11.2471
TkWRKY28	10.5403	14.6341	0.736686	14.5612	10.9437	15.0842
TkWRKY29	0.643699	0.99796	0.120515	2.12534	1.24355	0.639702
TkWRKY30	8.96497	5.62071	0.097526	9.87474	12.8396	9.7183
TkWRKY31	9.40394	9.63309	9.07138	13.1247	11.7991	6.99318
TkWRKY32	0.02285	0.084687	0	0.278015	0.02187	0.020901
TkWRKY33	0.388569	0.46225	0.450988	1.55745	0.220039	0.028657
TkWRKY34	0.013116	0.074795	0	3.96272	0.414623	0.02744
TkWRKY35	10.2934	12.5586	15.8768	16.674	14.8438	14.9549
TkWRKY36	0.822661	1.27941	9.43088	1.5932	2.04882	2.97459
TkWRKY37	38.5622	20.5905	0.305816	40.607	33.7904	6.19077
TkWRKY38	197.048	120.13	2.3103	43.1324	135.046	85.5722
TkWRKY39	0.233369	2.03803	0	0.017471	0.0854	0.259363
TkWRKY40	0.60542	0.370394	2.20275	3.31204	1.57794	1.18553
TkWRKY41	0.101093	0	0	5.76157	1.60302	0
TkWRKY42	0.042224	0.340283	0.435972	0.371221	0.151845	0.717741
TkWRKY43	1.70341	8.20773	2.20199	2.6753	2.21623	1.08536

TkWRKY44	0.084009	0.180658	1.00123	0.216328	0.030397	0.309564
TkWRKY45	0	0.076446	3.47733	0	0	0.035606
TkWRKY46	2.89292	3.8065	7.22938	4.41536	4.45116	3.99509
TkWRKY47	0.230898	0.34438	6.53236	0.218251	0.289326	0.893334
TkWRKY48	77.9903	83.3277	25.7757	35.1675	42.793	29.4103
TkWRKY49	10.9304	10.3557	1.4878	12.9326	11.7923	6.88214
TkWRKY50	6.53274	4.02417	0.532448	11.2082	28.1438	32.5843
TkWRKY51	0.711902	0.986585	0.238967	2.14765	1.84331	1.08823
TkWRKY52	6.69967	95.4851	1.36951	33.5655	22.0967	14.0501
TkWRKY53	0	0.032369	0.013525	1.6923	0.149391	0.173181
TkWRKY54	0	0.039217	0.046704	0.774748	0.057755	0.093086
TkWRKY55	0.089662	0	0	1.19065	0.513325	0.159201
TkWRKY56	40.9145	56.0356	21.1038	99.0179	98.0083	77.2679
TkWRKY57	0.765006	5.57521	2.1429	2.09201	3.16651	9.4029
TkWRKY58	2.1868	19.333	4.26992	4.23867	3.83539	8.32199
TkWRKY59	6.82959	10.6442	0.012767	10.0791	10.1933	7.80811
TkWRKY60	22.102	69.465	17.0546	17.1535	18.2569	27.7554
TkWRKY61	0	0	0	0.212326	0.039873	0
TkWRKY62	16.4222	6.3412	0.215222	70.6201	121.064	101.899
TkWRKY63	91.0756	120.887	9.29633	140.218	176.012	153.679
TkWRKY64	226.603	91.2839	50.0428	112.632	182.601	98.992
TkWRKY65	48.6155	14.9431	0.189324	53.1711	69.256	26.6543
TkWRKY66	29.9317	24.7035	1.80759	27.0487	31.3961	24.7135
TkWRKY67	13.7576	19.6653	13.5606	10.1792	13.6959	14.8603
TkWRKY68	0.689443	0.143333	0.703647	3.55371	1.27099	0.526074
TkWRKY69	0.35107	0.274724	0.030515	2.9786	0.553112	0.609663
TkWRKY70	8.79878	39.308	0.244617	19.6802	50.2113	50.8552
TkWRKY71	206.132	208.295	8.04607	393.543	373.731	322.925
TkWRKY72	1.17687	0.452387	0.0185	8.84855	5.82644	5.3123
