# Supplementary material for: Identification of the WRKY Gene Family and Characterization of Stress-Responsive Genes in Taraxacum kok-saghyz Rodin
Source: Int J Mol Sci. 2022 Sep 7;23(18):10270. doi: 10.3390/ijms231810270 (PMC9499643; doi:10.3390/ijms231810270)
Supplement: Supplementary file 1 [file ijms-23-10270-s001.zip › Table S6 Primers for Real-time RT-PCR.pdf]

Table S6 Primers for Real-time RT-PCR.

Primer name	Primer Sequence	Primer name	Primer Sequence
Q*TkWRKY4*-F	CAGCTACAGATTCATCCTCCCC	Q*TkWRKY4*-R	TTATTTGCAGCCTCGTTTGACG
Q*TkWRKY6*-F	ATCGGATGTTCCAGTTGACCAA	Q*TkWRKY6*-R	TAGAAGTAGAAGGCGCGTTTGT
Q*TkWRKY10*-F	TCCTGTTGGCCTGAGAGTTTAC	Q*TkWRKY10*-R	AAGATCTGCTTCACCACATCGT
Q*TkWRKY13*-F	GGTTAAGTCGATACGTGAGCCA	Q*TkWRKY13*-R	CCTGGGATTAGGGTTGCTTCTT
Q*TkWRKY15*-F	AACTCCAAATCCCCAAGGTGTT	Q*TkWRKY15*-R	GCAAGTGTGGTGGCCAAAATAT
Q*TkWRKY18*-F	AATGGTGGTCAACATCCGTACA	Q*TkWRKY18*-R	CTTCGGGTTCTTCCTTAGCACT
Q*TkWRKY21*-F	GGCTAAGACTCCATTTGGACCA	Q*TkWRKY21*-R	TCTTTATGGTGGATTGTGGGGG
Q*TkWRKY23*-F	CAGCTACAGATTCATCCTCCCC	Q*TkWRKY23*-R	TTATTTGCAGCCTCGTTTGACG
Q*TkWRKY27*-F	GACTCAGGCGAAAGTGAGAAGA	Q*TkWRKY27*-R	TAGCACCTTGGAAATGTAGCGT
Q*TkWRKY28*-F	ATGGTAAGGAGGTGATCATGGC	Q*TkWRKY28*-R	TCTCCATCTATAGCCGTCGTCA
Q*TkWRKY38*-F	ACTGGAAGAATCTCAGCAGCAA	Q*TkWRKY38*-R	TGGAACTGAGATGGGTGTTCTG
Q*TkWRKY39*-F	CAGCTAGTGCAACATCAACACC	Q*TkWRKY39*-R	TGTGTTCTTCTCCGAGTTCGTT
Q*TkWRKY56*-F	ACCAGTAATCAACCGGTTGTGT	Q*TkWRKY56*-R	GGTTTACCACCAGCAGATAGCT
Q*TkWRKY71*-F	TAACTCCAAGAAACGGAAGCCA	Q*TkWRKY71*-R	AGGTGTTGTTCTTGTCTGGGTT
GAPDH-F	AGTTGGTTTCGTGGTATGAC	*GAPDH*-R	ACATGTCAGTGAACAGGTAGAC
β-actin-F	GGAAGGATCTTTATGGGAAC	*β-actin*-R	CAGACACTATACTTCCTCTCAGG
